# Avian Species and Functional Diversity in Agricultural Landscapes: Does Landscape Heterogeneity Matter?

**DOI:** 10.1371/journal.pone.0170540

**Published:** 2017-01-26

**Authors:** Myung-Bok Lee, James A. Martin

**Affiliations:** 1 Department of Wildlife, Fisheries, and Aquaculture, Mississippi State University, Mississippi State, Mississippi State, United States of America; 2 Warnell School of Forestry and Natural Resources, Savannah River Ecology Lab, University of Georgia, Athens, Georgia, United States of America; Consejo Superior de Investigaciones Cientificas, SPAIN

## Abstract

While the positive relationship between avian diversity and habitat heterogeneity is widely accepted, it is primarily based on observed species richness without accounting for imperfect detection. Other facets of diversity such as functional diversity are also rarely explored. We investigated the avian diversity-landscape heterogeneity relationship in agricultural landscapes by considering two aspects of diversity: taxonomic diversity (species richness) estimated from a multi-species dynamic occupancy model, and functional diversity (functional evenness [FEve] and divergence [FDiv]) based on traits of occurring species. We also assessed how agricultural lands enrolled in a conservation program managed on behalf of declining early successional bird species (hereafter CP38 fields, an agri-environment scheme) influenced avian diversity. We analyzed breeding bird data collected at CP38 fields in Mississippi, USA, during 2010–2012, and two principal components of environmental variables: a gradient of heterogeneity (Shannon’s landscape diversity index) and of the amount of CP38 fields (percent cover of CP38 fields; CP38). FEve did not show significant responses to environmental variables, whereas FDiv responded positively to heterogeneity and negatively to CP38. However, most FDiv values did not significantly differ from random expectations along an environmental gradient. When there was a significant difference, FDiv was lower than that expected. Unlike functional diversity, species richness showed a clear pattern. Species richness increased with increasing landscape heterogeneity but decreased with increasing amounts of CP38 fields. Only one species responded negatively to heterogeneity and positively to CP38. Our results suggest that the relationships between avian diversity and landscape heterogeneity may vary depending on the aspect of diversity considered: strong positive effects of heterogeneity on taxonomic diversity, but weakly positive or non-significant effects on functional diversity. Our results also indicate that effectiveness of CP38 in conserving avian diversity, particularly, taxonomic diversity, could be limited without the consideration of landscape heterogeneity.

## Introduction

The relationship between diversity (i.e., biodiversity or species diversity) and habitat heterogeneity, e.g., heterogeneous vegetation structure at the local scale or heterogeneous habitat type at the landscape scale, is one of the most widely studied in ecology. Heterogeneous habitats are assumed to provide more niches or complementary resources and thus increase diversity of animals and plants [1, 2]. Although several recent studies suggest that the relationship can be non-linear [3–5], the positive relationship between diversity and habitat heterogeneity at local and landscape scales is generally accepted, particularly in agricultural landscapes [6–9].

Species richness is commonly used as a surrogate of diversity to examine the diversity-habitat heterogeneity relationship. However, richness is calculated based on observed richness by summing or averaging species found during surveys without accounting for imperfect detection (i.e., a species may not be detected even if present at a site). It is well recognized that ignoring imperfect detection for individuals and species can mislead inferences about species-habitat relationships and lead to biased estimates of occupancy, abundance, and richness [10–12]. In particular, incorporating detection probability (i.e., the probability of detecting species when it is present at a site) in the estimation of richness is crucial when the community is composed of large numbers of infrequently detected species [13, 14], as is often the case with avian communities. In recent years, several hierarchical models have been developed to estimate richness while accounting for heterogeneous detection probabilities among species. These models also produce species-level responses to environmental variables that cannot be obtained in single- species models. Although these models have been applied to evaluate effects of fragmentation, conservation management, and anthropogenic disturbance on richness [14–18], they are rarely used to assess the diversity-heterogeneity relationship. The species diversity-heterogeneity relationship can be more properly depicted with hierarchical multi-species models because such models provide relatively unbiased estimates of richness and species-level responses.

Moreover, richness represents only one aspect of diversity. While most inferences about the diversity-heterogeneity relationship are based on taxonomic diversity (mainly richness and the Shannon index), functional diversity has received less attention. Functional diversity quantifies the distribution and range of organismal traits (morphological, physiological, behavioral, or phenological traits) influencing ecosystem functioning or species’ responses to environmental conditions in a community [19, 20]. Functional diversity goes beyond guild classifications because it can deal with multiple traits simultaneously. Trait-based measures of diversity such as functional diversity have been recognized as alternatives that complement traditional diversity measures such as richness [21–25]. There is a growing consensus that the use of functional diversity combined with taxonomic diversity can improve our understanding of the interactions between aspects of biodiversity and environmental constraints [23]. Functional diversity is considered an important descriptor of ecosystem-level processes and of the effects of disturbances on ecosystem services or biodiversity [25–30]. Functional diversity indices are also often used to reveal community assembly processes (environmental filtering and competition or limiting similarity) by adopting a null model approach [23, 31–33]. Environmental filtering is assumed to play an important role in structuring a community when functional diversity is lower than the expected value obtained from random communities (i.e., species in a community are more functionally similar to each other than random expectation). Conversely, higher functional diversity than expected is recognized as evidence for limiting similarity, allowing the coexistence of functionally dissimilar species. Previous studies have demonstrated a decline in functional diversity for an array of taxa (birds, mammals, plants, and insects) and evidence of environmental filtering in agricultural landscapes [27, 32, 34].

Although various functional diversity indices have been proposed, many are redundant and highly correlated with species richness [23, 35]. Recent studies show that functional evenness (FEve) and functional divergence (FDiv) are not only relatively independent of other indices [23] but also better multi-trait indices for analyzing ecosystem functioning [36]. FEve defines the evenness of species abundance in functional space [37, 38]. FEve is low when abundance is unevenly distributed among species or functional distances among species are irregular. FDiv quantifies how the most abundant species are distributed within the volume of functional space [37, 38]. It also measures the degree of niche differentiation in a community [37]. FDiv decreases as the functional traits of the most abundant species are close to the center of the trait space.

We investigated the relationship between avian diversity and landscape heterogeneity (habitat heterogeneity at a landscape scale) in agricultural landscapes by considering functional diversity (FEve and FDiv) and taxonomic diversity (species richness). We calculated functional diversity based on traits of occurring species (observed richness, hereafter), whereas we estimated taxonomic diversity by accounting for heterogeneous detection probability among species. We were primarily interested in how landscape heterogeneity, such as the diversity of non-crop vegetation land covers or natural and semi-natural habitat types, influences avian diversity and individual avian species on Conservation Practice (CP38) fields, former production agricultural lands that have been restored to semi-grassland habitat. These CP38 fields are enrolled in a federal conservation program (similar to an agri-environment scheme in other countries) and are managed for early successional/grassland avian species (e.g., Northern Bobwhite [*Colinus virginianus*]; S1 Table) in Mississippi, USA. Our secondary objective was to assess how the amount of CP38 fields affects avian species diversity, about which little is known. We expected that landscape heterogeneity would increase both functional diversity and taxonomic diversity because heterogeneous habitats in a landscape can provide more niches or complementary/supplementary resources for a wider range of species’ traits. However, FEve and FDiv may behave differently. For instance, species richness is expected to increase as heterogeneity increases. If species added to the community have unique traits, functional divergence will increase, whereas functional evenness may not show a significant change, or even decrease, depending upon the distribution of new species’ abundances across the trait space. We also expected that while an increasing amount of CP38 fields influences the probability of occupancy by early successional/grassland species positively, both taxonomic and functional diversity may decrease because a high amount of CP38 fields could reduce landscape heterogeneity.

## Methods

### Ethics statement

**Fig 1:**
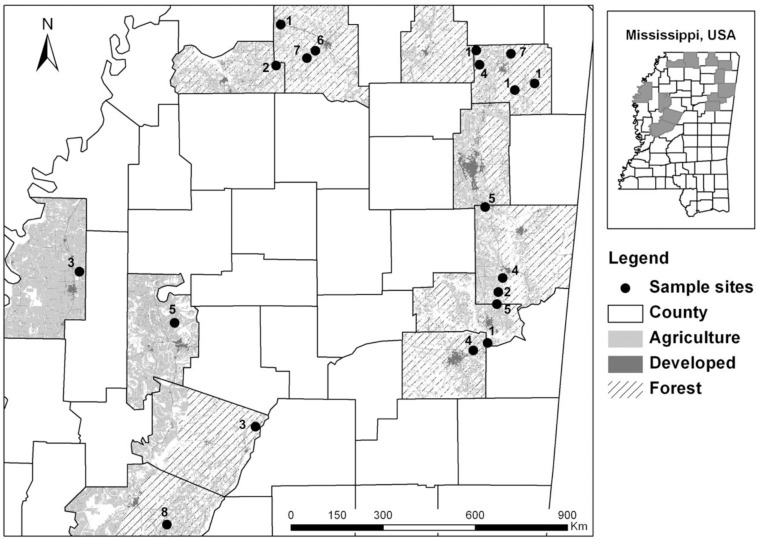
The Land cover and location of study regions surveyed in Mississippi, USA during 2010–2012. The numbers on the map represent the number of sample points (average 0.52 km apart, range from 0.2 to 1 km).

All field data used in our study were collected based on direct observation of birds, i.e., counting birds by sight and sound; see “Bird surveys and data” below. We confirm that our study did not involve endangered species or the handling of any animal. This type of observational study does not require approval from an Institutional Animal Care and Use Committee or equivalent animal ethics committee, thus no approval was sought. We also confirm that our study did not involve protected areas as it was performed on agricultural lands (See Fig 1 for location), and we acquired permission from land owners to access their land.

### Study sites

The study was conducted on CP38 fields located along an agriculture-forest gradient in northern Mississippi, USA (Fig 1). Conservation Practice 38 is a new initiative offered by the United States Department of Agriculture (USDA) Farm Service Agency that allows states to design conservation programs based on their own wildlife conservation needs. In Mississippi, CP38 aims to restore native grassland habitats for early successional/grassland species (S1 Table), particularly Northern Bobwhite but also other species such as Dickcissel (*Spiza americana*) and Eastern Meadowlark (*Sturnella magna*), by establishing native warm-season grasses, forbs, legumes, and shrubs on formerly agricultural lands. Production agricultural lands such as rowcrop and pasture/hay fields are prevalent across the study region. Forest is also dominant in some areas (Fig 1).

We selected a random sample of CP38 fields from the USDA Farm Service Agency contract database, resulting in 70 sample fields (Fig 1). One point was established at the center of each field or > 100 m from an edge.

### Bird surveys and data

Bird surveys were conducted by the same observer at sample points from mid-May through June each year during 2010–2012, using the CP38E-SAFE Mississippi monitoring protocol which was developed from the Conservation Program 33 monitoring protocol [39]. All points were surveyed once in 2010 and twice during 2011–2012. At each point, the observer recorded all birds detected within a 500 m radius of a sample point during a 10-min period. Care was taken to avoid double counting the same bird during the period. Each survey was performed between sunrise and 10:00. Surveys were not conducted during periods of rain or high wind.

A total of 76 species were detected at least once during 2010–2012; however, we excluded flyovers (birds only passing over the area via flight), waterfowl, nocturnal species, and raptors. Additionally, species that were detected on < 5% of the points were excluded; thus we used 46 species for analyses (See S1 Table for species list).

### Landscape data and environmental variables

To generate landscape variables, we digitized the satellite imagery from 2010 National Agriculture Imagery Program and the world imagery base map available in ArcGIS version 10. We classified land cover into 8 classes: production agriculture (rowcrop and pasture/hay field), CP38 field, pine forest, non-pine forest (hardwood forest and mixed forest), shrubland, open habitat (early successional vegetation and open-forest, > 50% open canopy cover of any forests), built-up/barren (house, road, and bare ground), and open water (mostly pond) (Table 1). In our study, areas classified as rowcrop field and pasture/hay field were production agricultural lands and they were not enrolled in any conservation program. We calculated the percent cover of each class within a 1.5 km radius surrounding a sample point using FRAGSTATS. We chose a 1.5 km radius large enough to encompass different matrices surrounding a CP38 field, given that the size of several CP38 fields wass > 50 ha and the total size of closely located CP38 fields was > 100 ha.

**Table 1:** Summary statistics of land covers in a 1.5 km radius area surrounding a sample point.

Type	Mean % (standard deviation)	Min%	Max%
Production agriculture	38.4 (14.6)	9.20	66.1
CP38 field	7.3 (4.4)	1.2	19.8
Pine forest	11.9 (8.8)	0.0	35.1
Non-pine forest	26.6 (12)	5.5	54.4
Shrubland	2.3 (4.1)	0.0	26.9
Open habitat	7.1 (6.0)	1.0	30.3
Built-up/barren	5.1 (4.9)	0.4	25.7
Open water	1.5 (1.4)	0.0	5.4

Open-forest (> 50% open canopy cover of any forest but mostly pine forest) and early successional herbaceous vegetation were grouped into open habitat because both classes function similarly and the amount of open-forest was very low.

We used Shannon’s Diversity Index (SHDI) to represent landscape heterogeneity. SHDI is calculated based on the number of non-crop vegetation cover types (which can be referred to as natural and semi-natural habitat) including CP38 field, pine-forest, non-pine forest, shrubland, and open habitat class, and their evenness within a landscape. We acknowledge that we focused on the compositional heterogeneity of the landscape. We did not include configurational heterogeneity because configuration indices such as interspersion/juxtaposition index and contagion index were correlated with SHDI in our study (Pearson correlation, r = 0.62 and r = -0.97, respectively).

For environmental variables, we included SHDI, percent cover of CP38 fields, and percent cover of production agricultural land use (rowcrop and pasture/hay field) to account for a gradient of agricultural land that is a dominant anthropogenic land use across our study region. However, percent cover of production agricultural land and SHDI were highly correlated (r = -0.72). We performed a principal component analysis on those three variables to identify independent patterns of environmental variation within our data set and to avoid statistical multicollinearity. We retained two principal components with eigenvalues ≥ 1, capturing 91% of the total variation: heterogeneity, a gradient from agricultural lands to increasing SHDI; and CP38, a gradient of increasing the amount of CP38 fields (i.e., percent cover of CP38 fields) within a landscape. Those components were used as environmental variables in all analyses.

### Functional diversity

To characterize functional diversity, we used traits known to be functionally important in other studies [27, 29, 30, 31, 40, 41]: one continuous trait type—body mass—and 11 binaries of three trait types—diet, foraging behavior and location—and migratory status (Table 2 and see S1 Table for detail description). All traits but migratory status are strongly associated with resource use. We compiled data on all traits from The Birds of North America online database (BNA, [42]) and Ehrlich *et al*. [43]. Missing body mass data from BNA were compiled from Dunning [44]. We used species observed (not from the hierarchical model below) to calculate functional diversity. We did not incorporate imperfect detection into the calculation of functional diversity because there is no available method to do so. We pooled 3 years (2010–2012) of survey data and considered a species as detected if it was observed at least once during the surveys. For abundance, we used the maximum number of individuals observed.

**Table 2:** Traits used for the estimation of functional diversity indices.

Trait type	Trait categories	Value type of each trait category
Body mass	Body mass	Continuous
Diet	Insects, seeds/grains, various items (omnivorous), others	Categorical
Foraging (behavior and location)	Bark gleaning, foliage gleaning, ground foraging, hawking, sallying, others	Categorical
Migratory status	Migrant	Binary

We used FEve and FDiv as functional diversity indices and calculated their values at each point following a common approach employed in other studies [30, 31, 35, 38]. We first constructed a trait matrix for 46 species and converted it into a dissimilarity matrix, the Gower dissimilarity matrix [45]. Using the dissimilarity matrix, we performed a principal coordinate analysis (PCoA). We selected four PCoA axes that explained 76% of the variation in the distance matrix. Those PCoA axes were adopted as new traits to estimate the values of functional diversity indices. The number of PCoA axes defines the number of dimensions of functional space, which in turn affects the measurement of functional diversity. To determine the quality of functional spaces represented by four PCoA axes, we calculated the mean squared deviation (mSD) following the method proposed by Maire et al. [46]. The mSD assesses the degree of inconsistency between initial and final functional distances: as mSD is close to 0, the quality of functional space is high. The mSD of the first four (0.0017) or five (0.0016) PCoA axes was lower than the mSD of other PCoA axes (S1a Fig). The scree plot also showed a break between the fourth and fifth components or axes, indicating that components after the fourth accounted for a trivial amount of variance in our data (S1b Fig). Given these patterns, we are confident that four PCoA axes chosen for our study represent the functional distance between species appropriately and thus any bias associated with the choice is minimal. We used InfoStat (version 2012, [47]) to create a distance matrix and to perform the PCoA and the software program FDiversity [48] to calculate the functional diversity indices.

We adopted a null model approach of community data matrix randomization and calculated the standardized effect size [49, 50] at each sample point or community to assess whether changes in observed functional diversity are dominated by changes in species richness. A null model approach is recommended for analysis utilizing functional diversity measures to make inference, particularly, when those measures are correlated with species richness [49]. Comparison between observed values and expected values calculated from random communities is also commonly used to examine community assembly processes. In our data, species richness was moderately correlated with FDiv (Pearson’s correlation r = 0.408, *P* < 0.001) but not with FEve (r = 0.014, *P* = 0.91); thus, we applied the null model approach to FDiv. We simulated 999 communities by randomly choosing species from the species pool (all species found at any point) while maintaining species richness as constant (i.e., the same as observed richness) at each point. Abundance of each species was also randomly selected, and the pattern of abundance remained constant within a point. This randomization was carried out using the picante package in R. The deviation in observed FDiv from expected FDiv was measured based on the standardized effect size, FDiv.SES = (Obs − Exp_mean_)/Exp_sd_, where Obs is the observed FDiv value and Exp_mean_ and Exp_sd_ stand for the mean and the standard deviation of expected FDiv values (FDiv values calculated from 999 random communities). FDiv.SES values below -1.96 (the lower confidence bound) or above +1.96 (the upper confidence bound) were considered statistically significant at *P* < 0.05 [50]. Significantly low and high FDiv.SES indicates the evidence for environmental filtering and limiting similarity, respectively.

The relationship between functional diversity and two environmental variables was analyzed with a linear regression model, using FEve and FDiv as response variables. We also used null models to test the non-randomness of the overall trend in the relationship between FDiv and environmental variables, i.e., whether the relationship differed from random expectations. We simulated the linear regression model of FDiv 999 times with the FDiv value generated from each of 999 random communities as a response variable for each simulation. Then, parameter estimates (regression coefficients) from an empirical model were compared with parameter estimates from the null models at α = 0.05 (two-tailed test). We determined spatial autocorrelation by constructing Moran’s I correlograms of the regression model residuals in SAM 4.0 (Spatial Analysis in Macroecology, [51]). We did not find significant spatial autocorrelation (P > 0.05) in the residual of any response variable.

### Richness and species-level responses

We adopted the hierarchical modelling framework of a multi-species occupancy model [13, 14] and a multi-season (dynamic) occupancy model [15, 52, 53] to investigate the effects of heterogeneity and CP38 on species richness and individual species. These models require presence/absence (detection/non-detection) data from repeated surveys. They were developed to take into account imperfect detection by incorporating a detection probability into occupancy estimation. Multi-species occupancy models consider species as random effects governed by common community-level distributions and deal with heterogeneous detections among species. They also account for both species-level effects and overall effects of environmental covariates on the community. Multi-season occupancy models, in general, are applied to studies where surveys are conducted during multiple seasons/years with repeated visits within a season/year. They allow for changes in occupancy status at a site due to extinction/survival or colonization events between seasons/years. While we conducted two surveys per year during 2011–2012, we had only one survey in 2010. In open-population modelling, we found that the missing 2nd survey during the 1^st^ year resulted in overestimation of mean detection for the year. To minimize possible biases associated with a lack of repeated surveys, we did not include the 2010 data and used 2-year data collected during 2011–2012.

Before analyses, we examined Moran’s I correlograms of Pearson residuals from a logistic regression model including two environmental variables. Of the 46 species, the residuals of 15 were correlated. To account for the spatial dependency of occupancy status among sites, we calculated an autocovariate (Acov) by modifying Royle and Dorazio’s method ([13]; See S1 Appendix for details) and added Acov to the model as an additional covariate for those 15 species.

Our model follows the typical structure of hierarchical occupancy models, linking two regression models: an ecological process model estimating the true occupancy state (latent state) and an observation model estimating the observed occupancy state by combining the latent state and detection probability [53]. In the ecological process model, Z_*ijt*_ denotes the latent occupancy state of species *i* at site *j* during year *t* where Z_*ijt*_ = 1 if species *i* occupies site *j* during year *t* and Z_*ijt*_ = 0 otherwise. The latent occupancy state was modelled to be drawn from a binomial distribution, i.e., Z_*ijt*_ ~ binomial(ψ_*ijt*,_ 1), where ψ_*ijt*_ is the probability that species *i* occupies site *j* during year *t*. In the observation model, the observed occupancy state (y_*ijtk*_) was specified similarly, but as a product of the latent occupancy state and detection probability: y_*ijtk*_ ~ binomial(*p*_ijtk_*Z_*ijt*_, 1), where *p*_*ijtk*_ is the detection probability of species *i* at site *j*, year *t* and survey *k* and y_*ijtk*_ is 1 if species *i* is detected at site *j*, year *t* and survey *k* and y_*ijtk*_ = 0 otherwise.

We assumed the occupancy status of site *j* by species *i* could change between the two years. We adopted Russell et al.’s approach [15] that was derived from Royle and Kéry’s multi-season occupancy model [52], but we included two sets of parameters associated with survival (species remaining at a site from previous year) and colonization (species occupying a site, which was unoccupied the previous year) [18]. Occupied site *j* (Z_*ij1*_ = 1 and 1- Z_*ij1*_ = 0) by species *i* during year 1 can be re-occupied following year 2 with an effect of κ_*i*_ (a coefficient governing survival between years 1 and 2). Conversely, an unoccupied site *j* (Z_*ij1*_ = 0 and 1- Z_*ij1*_ = 1) by species *i* during year 1 can be occupied following year 2 with an effect of υ_*i*_ (a coefficient governing colonization between years 1 and 2). We used the logit link function for ψ_*ijt*_ and modelled as a linear combination of a species effect and site- and/or species-specific covariates (landscape heterogeneity, the amount of CP38, and autocovariate) as follows:

$$\text{ logit}\left(\psi_{ij1}\right)=u_{i}+\alpha 1_{i}{\;*\text{ Heterogeneity}}_{j}+\alpha 2_{i}\;*\text{ CP}38_{j}+\delta_{i}{\;*\text{ Acov}}_{ij}\;\text{for }t\;=\;1$$

and

$$\text{logit}\left(\psi_{ij2}\right)=u_{i}+\kappa_{i}{\;*\text{ Z}}_{ij1}+\upsilon_{i}\;*\;(1-{\text{Z}}_{ij1})+\alpha 1_{i}{\;*\text{ Heterogeneity}}_{j}+\alpha 2_{i}\;*\text{ CP}38_{j}+\delta_{i}{\;*\text{ Acov}}_{ij}\;\text{for }t=2$$

where *u*_*i*_ is a species-level intercept, the coefficients α1_*i*_, α2_*i*,_ and δ_*i*_ represent effects of the relevant covariates on the occupancy of species *i*, and κ_*i*_ and υ_*i*_ are survival and colonization effects between year 1 (*t* = 1) and year 2 (*t* = 2), respectively. The parameters u_i_, α1_i_, α2_i_, κ_*i*_, υ_*i*_ and δ_i_ are normally distributed species-specific random effects, for example, α1_i_ ~ *N*(μ_α1_, σ_α1_) and α2_i_ ~ *N*(μ_α2_, σ_α2_).

For the observation model, we assumed that detection probability varied by species (*v*_*i*_, species-level effect). The species-level effect was also modelled by incorporating a correlation (ρ) between species occurrence and detection to account for potential bias in detectability because abundant species are likely to be more easily detected and prevalent across the landscape as described in Royle and Dorazio [13] and Zipkin et al. [14] (See S1 Appendix for details). We did not include survey-specific, observer, or year effects because those effects on detection are likely negligible in our study given that the observer remained constant for all surveys, the time between surveys within a year did not vary greatly (< 21 days), and surveys were conducted during similar periods for both years. The detection probability was modelled as:

$$\text{logit}\left(p_{ijtk}\right)=v_{i}$$

where *v*_*i*_ is a species-level intercept and represents a species-specific random effect.

We conducted the analysis using the R2jags package in R. Model code is provided as S1 Appendix. We ran three parallel chains for 120,000 iterations, discarded the first 50,000 for burn-in, and thinned the posterior chains by 20. We also confirmed that our model converged and fit the data well based on the Gelman-Rubin statistic (Gelman-Rubin statistics, shrinking factor R < 1.1) and Bayesian *P*-value (Bayesian *P*-value = 0.52), respectively. Precision of parameter estimates was determined using the 95% Bayesian credible interval (BCI) which is similar to a 95% confidence interval.

## Results

### Functional diversity

FEve did not show a clear pattern with observed richness, confirming the independent relationship between FEve and observed richness. However, FEve tended to peak at the intermediate level of observed richness, though the trend was very weak (S2 Fig). FDiv showed a saturated pattern: it increased moderately with observed richness to a certain point and then flattened (S2 Fig). There was no significant correlation between expected FDiv and observed richness (r = -0.027, *P* > 0.05).

While FEve did not respond significantly to either heterogeneity or CP38, FDiv responded positively to landscape heterogeneity and negatively to the amount of CP38 fields (Table 3). FDiv increased as different types of non-crop vegetation land covers (pine forest, hardwood and mixed forest, open habitat, etc.) increased and percent cover of agricultural lands decreased within a landscape. It also decreased with increasing amount of CP38 fields (Table 3). Both regression coefficients (i.e., Heterogeneity and CP38) were significantly different from random expectations. Heterogeneity and CP38 from the empirical model were more positive (*P* < 0.001, Z-score = 3.303) and negative (*P* < 0.05, Z-score = -2.407) than coefficients of null models, respectively.

**Table 3:** The estimates of the environmental covariates from a regression model for functional evenness (FEve) and functional divergence (FDiv). Parameter estimates that significantly differed from simulated parameter estimates are in bold.

Indices	Estimate (standard error)		Adjusted R^2^
	Heterogeneity	CP38	
FEve	-0.004 (0.005)	0.000 (0.005)	0.022
FDiv	**0.020** (0.004)**	**-0.012** (0.004)*	0.361**

Significance:

*, P <0.05;

**, P < 0.001.

**Fig 2:**
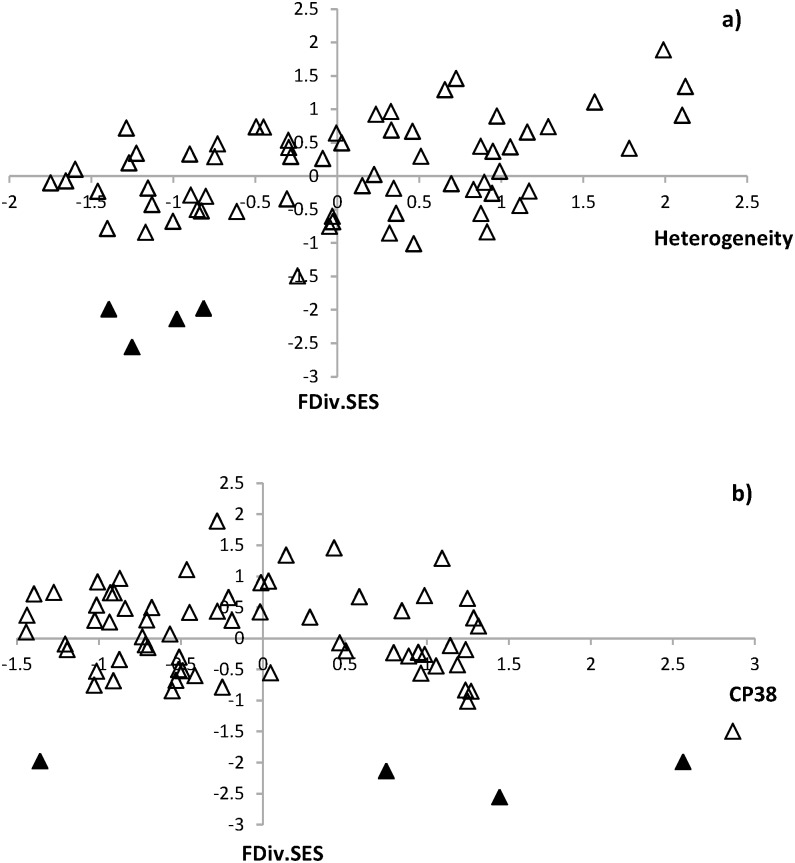
Variation in FDiv.SES along a gradient of landscape heterogeneity (a) and amount of CP38 fields (b) within a landscape. A black triangle indicates a significant FDiv.SES value.

However, observed values of FDiv were not notably different from expected values (mean values of 999 random communities) in most cases. Of 70 sample points, only 4 points showed a significant departure in observed FDiv from expected FDiv along a gradient of landscape heterogeneity (Fig 2a). FDiv.SES values of those 4 points were negative. All of the 4 points were located in the landscapes with a low level of heterogeneity and high level of agricultural land use (average percent cover of agricultural lands, 57.1 with a standard deviation of 7.7). In the case of CP38, significant FDiv.SES values were found at 4 points. Their FDiv.SES values were also negative and mostly located at the landscapes with moderate to high amount of CP38 fields (Fig 2b).

### Community (richness)- and species-level responses

The estimates (μ_α1_ and μ_α2_) of community-level hyper-parameters for heterogeneity and CP38 were positive and negative, respectively (Table 4). These estimates represent the mean effects of heterogeneity and CP38 on occupancy across all species in the community. These results suggest that the mean occupancy probability of species was greater when a CP38 field was embedded in a matrix composed of diverse non-crop vegetation versus an agriculture-dominated matrix. Conversely, the mean occupancy probability was low when the amount of CP38 fields was high. In summation, species richness increased with increasing landscape heterogeneity but decreased with increasing amount of CP38 fields in a landscape. The mean effect of CP38 was 1.53 times (0.43/0.28) greater than that of heterogeneity. The 95% Bayesian credible interval (BCI) of both estimates did not contain 0, indicating relatively constant responses across species in the community to those environmental variables.

**Table 4:** Summary of community-level hyper parameters for the occupancy covariates.

Hyper parameters		Mean	Standard deviation	95% BCI
Heterogeneity	μ_α1_	0.277	0.118	0.053–0.520
	σ _α1_	0.503	0.119	0.301–0.766
CP38	μ_α2_	-0.43	0.135	-0.710 –-0.176
	σ _α2_	0.623	0.142	0.383–0.941

μ_α1_ and μ_α2_ are the mean effects of landscape heterogeneity and of amount of CP38 fields (CP38) across all species, respectively. σ _α1_ and σ _α2_ represent the standard deviation of each variable among species.

There was a weak positive correlation between occurrence and detection (mean ρ = 0.24); however, a wide 95% BCI (-0.18–0.58) indicated large uncertainty in the correlation among species. In addition, the estimated richness from the model that accounted for variable detection probabilities among species was higher than observed richness (the number of species detected) (S3 Fig).

**Fig 3:**
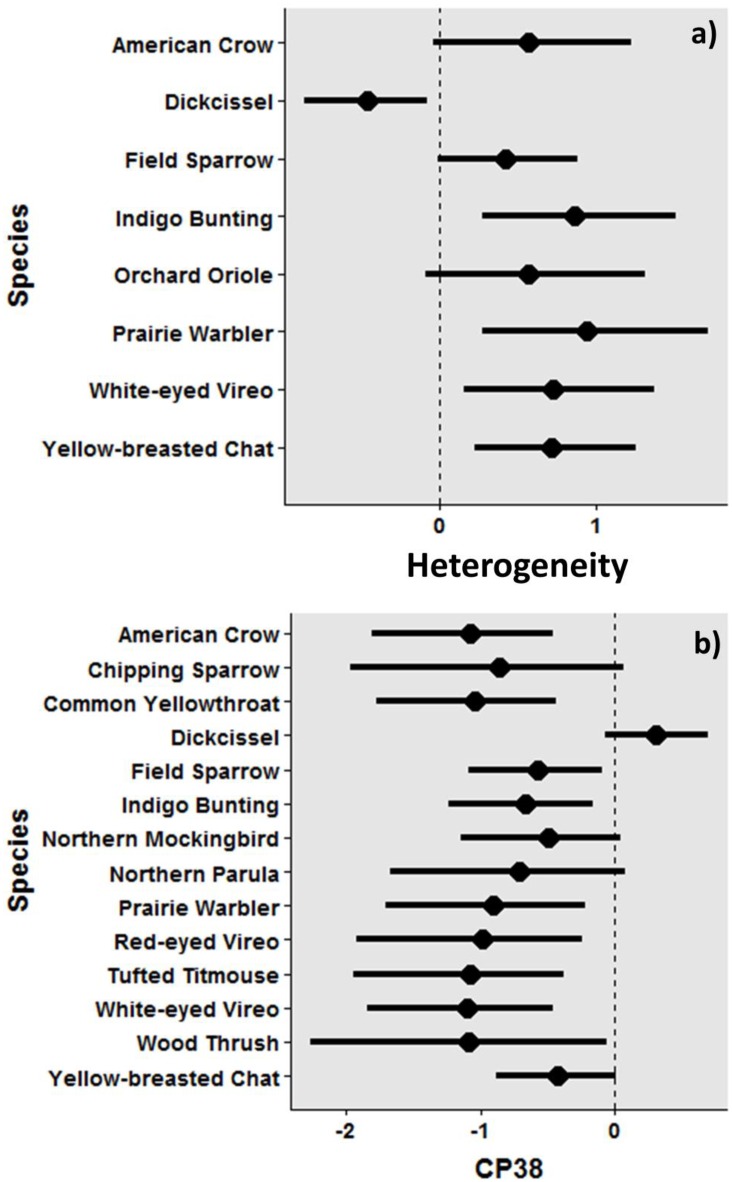
Species level responses to two environmental covariates, heterogeneity (a) and CP38 (b). Heterogeneity (landscape heterogeneity) represents a gradient from agricultural lands to increasing Shannon’s Diversity Index (SHDI); and CP38 represents a gradient of increasing the amount of managed fields (CP38) within a landscape that covers a 1.5 km radius area surrounding a sample point. All values were estimated from the multi-species dynamic occupancy model. The graph shows only the species with 95% Bayesian credible intervals (bars on the graph) that did not or only marginally contain 0. See S4 Fig for the complete responses of all species.

Species-level responses to environmental variables showed a similar pattern to the community-level responses. Eight species had 95% BCIs not containing 0, or only slightly overlapped 0, and all open-forest and shrub species (Indigo Bunting [*Passerina cyanea*], Orchard Oriole [*Icterus spurius*], Prairie Warbler [*Setophaga discolor*], White-eyed Vireo [*Vireo griseus*], Yellow-breasted Chat [*Icteria virens*]) showed positive responses to landscape heterogeneity (Fig 3a; S4 Fig for all species’ responses). The response of early successional/grassland species (Dickcissel and Field Sparrow [*Spizella pusilla*]) varied by species. While the probability of occupancy by Field Sparrow increased with heterogeneity, the probability of occupancy by Dickcissel decreased. Increasing the amount of CP38 fields in a landscape negatively influenced species occupancy and only Dickcissel showed a strong positive response (Fig 3b; S4 Fig for all species’ responses).

## Discussion

We found that the effect of landscape heterogeneity was strongly positive on taxonomic diversity (species richness) but weakly positive (FDiv) or non-significant (FEve) on functional diversity. While these results partly supported the hypothesis predicting the positive relationship between avian diversity and habitat heterogeneity, they indicated that taxonomic and functional diversity can respond differently to landscape heterogeneity. Negative responses of taxonomic diversity to the amount of CP38 fields suggested a need to consider landscape heterogeneity to enhance the effectiveness of CP38 as a community-level conservation program.

### Taxonomic diversity

Species richness increased with landscape heterogeneity defined by different non-crop vegetation cover, i.e., natural and semi-natural habitats within a landscape. At the species-level, the probability of occupancy for most species, especially open-forest and shrub species, also increased with landscape heterogeneity. These results supported the positive relationship between diversity and habitat heterogeneity that is widely observed in avian studies [1, 6, 54]. According to the habitat heterogeneity hypotheses [1, 2, 55], heterogeneous landscapes (landscapes composed of diverse vegetation cover types) can offer more niches or complementary resources, such as food and nest sites, than homogeneous landscapes, particularly where local heterogeneity is low due to intensive agricultural land uses. Landscape heterogeneity can also facilitate resource use by providing species with supplemental habitats containing resources required by the species (landscape supplementation [56]). Thus, diversity, which is commonly measured by species richness, is expected to increase through an accumulation of species related to the cover types and presence of species that require more than one cover type in a landscape as landscape heterogeneity increases [8]. For that reason, habitat heterogeneity at multiple spatial and temporal scales is often identified as a key factor in restoring or maintaining biodiversity in agricultural landscapes [6, 8]. Our study confirms the importance of heterogeneity of the surrounding landscapes to enhance taxonomic diversity in agriculture-dominant areas.

However, it should be pointed out that the positive diversity-habitat heterogeneity relationship may not be monotonic. Several recent studies describe tradeoffs between heterogeneity and the amount of effective area available per habitat type because increasing heterogeneity would decrease the amount of effective area, leading to a reduction in the population size that each habitat supports and consequently increases the risk of stochastic extinction [3–5]. High landscape heterogeneity can also increase habitat fragmentation per se and thus influence diversity negatively. Given the positive relationship observed in our study, the heterogeneity level does not seem to reach the break point that could generate negative responses. Most habitat types, except forest (pine and non-pine), may not have been abundant enough to support large populations because agricultural land use was so predominant. However, it is possible that more increases in heterogeneity can fragment even forest patches to a level that each patch is too small to support the local population and becomes functionally disconnected from adjacent patches. Further study is needed to examine the potential unimodal (humpback shaped curve) relationship between diversity and heterogeneity and define the break point of heterogeneity shifting the relationship from positive to negative.

We did not find strong positive effects of CP38 on species richness. The management of CP38 targets early successional/grassland avian species inhabiting grassland and open forest in agricultural landscapes (S1 Table). Although CP38 largely focuses on Northern Bobwhite as a representative species of early successional/grassland birds in Mississippi, it also considers other species such as Dickcissel, Eastern Meadowlark, and Prairie Warbler. Given that the occupancy by Dickcissel (grassland species) increased with the amount of CP38 fields, our results indicate that CP38 can be beneficial for some early successional/grassland species. This Dickcissel response is consistent with other studies that assessed the effects of Conserve Reserve Programs (CRP, including CP38 and other similar practices) on grassland birds [39, 57]. While negative responses of forest interior species (e.g., Northern Parula [*Parula americana*], Red-eyed Vireo [*Vireo olivaceus*], Tufted Titmouse [*Baeolophus bicolor*], Wood Thrush [*Hylocichla mustelina*]) are not surprising, non-significant or negative responses of other early successional/grassland species and open-forest species (e.g., Eastern Meadowlark, Field Sparrow, Northern Bobwhite, Indigo Bunting, Prairie Warbler) may raise a question about the effectiveness of CP38 as a community-level conservation program. Many studies have documented variable effects of CRP on avian species: the effect can be positive, negative, or neutral, depending on species, landscapes, and regions [57–59].

CP38 fields in our study have been managed for only 2–3 years. This relatively short history of management may not have notably improved the habitat quality of CP38 fields, which may make CP38 fields indistinguishable from surrounding agricultural lands, especially, pasture/hay fields (i.e., low ecological contrast, [60]). More time may have been needed for other species to colonize these newly created fields. Thus, our results should not be taken as evidence of the ineffectiveness of CP38, but as an indication that the effectiveness of current CP38 management for avian conservation beyond several early successional/grassland species can be limited without incorporating information about the heterogeneity of surrounding landscapes.

Our study adopted a recently developed hierarchical multi-species model to investigate the relationship between richness and heterogeneity. The relatively greater richness estimates compared to observed richness could be a result of taking into account variable detection probabilities among species in the model. However, we note that while the hierarchical multi-species model enables us to observe responses of each of 46 species, parameter estimates of some infrequently detected species (e.g., American Goldfinch, [*Spinus tristis*], Brown Thrasher [*Toxostoma rufum*], and Ruby-throated Hummingbird [*Archilochus colubris*]) exhibited large uncertainty (wide 95% BCI). This result suggests that sparse detection data can still be an issue as reported in other studies [15, 17] and intensive single-species monitoring efforts may be required for those species to reduce uncertainty, especially where they are of interest to management [15].

### Functional diversity

Unlike taxonomic diversity, responses of functional diversity to the two environmental variables were subtle. FEve, which was independent of species richness, did not show a clear pattern with varying landscape heterogeneity or the amount of CP38 fields. FEve may indicate under/over utilization of resources available to species [37]. Resources would be more efficiently used in a community with high FEve because the distribution of the abundance of each species is relatively even throughout the trait space. Resources would be underexploited in a community with low FEve, and this may make the community more susceptible to invasion (empty niche hypothesis [61]). Non-significant response of FEve in our study suggests that landscape heterogeneity and the amount of CP38 fields do not have a strong impact on the regularity of spacing between species in trait space and efficiency in resource utilization.

Responses of FDiv were similar to species richness: positive to landscape heterogeneity and negative to the amount of CP38 fields. The most abundant species are farther from the center of trait space in heterogeneous landscapes where agricultural land use is relatively low and in landscapes where the amount of CP38 fields is low. Significant differences between parameter estimates from an empirical model and from null models also indicated non-random patterns in the responses of FDiv. However, based on FDiv.SES at each sample point, a majority of FDiv values were consistent with random expectations, suggesting that to some degree the variation in FDiv is influenced by changes in species richness. Although most FDiv values did not differ from expected values, it is noteworthy that all significant departures from expected FDiv (4 of 70 sample points) were negative (i.e., FDiv.SES values were below the lower confidence bound) and found in relatively homogeneous, agriculture dominant landscapes. Lower functional diversity compared to random expectation is generally considered as an evidence for environmental filtering in structuring communities. Anthropogenic land use is known to influence the strength of environmental filtering [62]. Although there is some inconsistency in the strength among indices used in previous studies, most studies show the increasing role of environmental filtering and decline in functional diversity for an array of taxa as the intensity of agricultural land use increases, largely due to disturbances created by agricultural practices (non-crop vegetation removal, cropping, grazing, etc.) and simplified habitat structure and composition [23, 27, 32, 63]. The pattern observed in our study is somewhat congruent with the findings of previous studies, implying that environmental conditions constrain traits occurring in a bird community in our study region where production agricultural land-use is dominant. However, it is also noticeable that FDiv.SES values tended to shift slowly from negative to positive with increasing heterogeneity. Although all positive departures were non-significant, the trend might be an indication of potential niche differentiation and increasing strength of limiting similarity in heterogeneous landscapes. To test this possibility, further investigation using an increased number of sites representing wide heterogeneity is needed.

As for CP38, significantly lower FDiv, in 3 of 4 significant cases, were found at landscapes with moderate to high amount of CP38 fields. While overall effect of the amount of CP38 fields on FDiv could be trivial (i.e., differences between FDiv and expected values were non-significant across sample points), this result may support, as discussed previously, the possibility of poor habitat quality of CP38 fields for avian communities, which may result in a trivial difference in quality between CP38 fields and surrounding agricultural lands as a habitat for birds.

FDiv showed a positive correlation with species richness at low richness, but flattened after a certain point. Moderate correlation of FDiv with species richness is contrary to the theoretical expectation (no correlation) from Villéger *et al*. [38] and Mouchet *et al*. [23]; however, different relationships (saturating or negative relationship) between functional diversity indices and species richness have been observed in other studies [31, 32, 64]. The saturating pattern with increasing richness indicates that bird communities with low richness are likely composed of functionally unique species, but as richness increases, new species added to the community could be more functionally redundant [30].

Considering the overall non-significant deviations of functional diversity values from random expectations and the saturating relationship between FDiv and species richness, one may conclude that landscape heterogeneity did not have a positive effect on functional diversity but only on taxonomic diversity. However, we observed significantly low FDiv only at low levels of heterogeneity (i.e., high levels of agricultural land uses) and the tendency of increasing FDiv with heterogeneity. This pattern indicates that in a predominantly agricultural landscape, a slight increase in heterogeneity can enhance both species richness and functional diversity. In that sense, landscape heterogeneity may have some positive effect on functional diversity but its effect is not as strong as on species richness. Given that the positive relationship between avian functional diversity and landscape heterogeneity has been rarely tested empirically, there are few studies for comparison. Recently, Sittes *et al*. [65] found that in fire-prone system, environmental heterogeneity (heterogeneous vegetation composition and structure) enhanced by fire increased functional diversity. However, Sitter *et al*. considered more traits (6 types) and levels (~ 40 binary categories) than ours. The value of functional diversity can be strongly influenced by the type and number of traits chosen for a study [27]. We used four traits that are commonly used in other studies and known to be important for birds regarding resource use and acquisition. However, one category in diet type is predominantly shared by most species in our study. More than 90% of species prey upon insects to some degree in their life cycle, particularly, during breeding season, although 40% of those species also feed upon other items (seeds, invertebrates, etc.; S1 Table). Relatively small variations in the diet type might obscure the relationship between functional diversity and landscape heterogeneity in our study.

### Limitation

Our results demonstrate varying relationships between avian diversity and environmental variables; however, there are several caveats that may affect the strength of inference. First, we used a 1.5 km landscape size which is close to the common scale (1 km landscape size) used in numerous avian studies. However, significant avian species-environmental relationships can occur at larger scales (e.g., 5 km landscape size; [66]). Species’ responses to landscape features (composition vs. configuration) or habitat heterogeneity can be scale-dependent [67, 68]. A recent study also demonstrated that the association of anthropogenic land use with functional diversity and taxonomic diversity can change with scale [69]. Due to logistical constraints associated with producing land cover data by digitization and the low accuracy of available land cover data, we could not employ a multiple scale approach to verify the adequacy of the scale or test possible effects of variation in heterogeneity at larger scales. Thus, our inferences about the diversity-heterogeneity relationship may be limited to the scale used in our study rather than generalized across all scales. Second, while our study focused on compositional heterogeneity (SHDI), configurational heterogeneity (complexity of spatial arrangements among patches) can also impact the diversity-heterogeneity relationship given the well-known effects of landscape configuration on population persistence, dispersal, and movement behavior [8, 70]. We excluded configurational heterogeneity due to the correlation between configurational metrics and SHDI. Although these correlations cause a multicollinearity issue and difficulty in assessing the relative effects of compositional and configurational components of landscapes, landscape heterogeneity can be described comprehensively with a combination of both components [8]. Thus, landscape heterogeneity in our study may reflect one aspect of actual landscape heterogeneity due to the exclusion of configurational heterogeneity. Last, we adopted a focal patch sampling approach [71] with CP38 field as the focal patch. While this is a common approach in landscape research, it cannot fully depict properties of a whole landscape and their influences on species, assemblage, and ecological processes [72, 73]. A sampling design including multiple habitats within a landscape is considered more suitable for the assessment of landscape-level biodiversity; however, it is rarely adopted because of the intensive sampling efforts required [72, 74]. Although the relatively large-sized area (500 m radius) used for avian point counts likely alleviates the limitation of a focal sampling approach, our inference may be constrained to local-level biodiversity focused on a single habitat type (CP38 field).

## Conclusion

Our study shows that the positive relationship between avian diversity and landscape heterogeneity, which is often assumed in agricultural landscapes, can vary depending on the aspect of diversity explored. While the number of species in a community increases as landscape heterogeneity increases, functional traits of the species newly added to the community may not significantly differ from those of species already present in the community. That is, landscape heterogeneity has a strong positive effect on taxonomic diversity (species richness), whereas its influence on functional diversity can be weakly positive or non-significant. These patterns highlight the importance of considering multiple aspects of diversity to comprehensively understand the relationship between diversity and environmental constraints. Our study also suggests that future implementation of conservation measures in agricultural landscapes should consider the landscape context to assure maximum efficacy and perhaps reduce negative impacts to non-targeted species. Adopting a hierarchical multi-species model can improve inference by providing species-level responses and community-level responses. We emphasize a need for future studies investigating the responses of richness and functional diversity to compositional and configurational heterogeneity at different scales. A model that incorporates detection probability into the calculation of functional diversity indices is needed and may promote accuracy of those indices and subsequent inference.

## Supporting Information

S1 Fig The mean squared deviation (mSD) with varying numbers of dimensions (i.e., PCoA axes) of functional spaces and the scree plot showing the eigenvalue of each PCoA axis. (PDF)

S2 Fig Relationship between species richness and (a) FEve and (b) FDvi. Expected FDiv is the mean FDiv value obtained from 999 random communities.
(PDF)

S3 Fig Comparison of mean estimated richness (resulting from a hierarchical multi-species dynamic occupancy model) and mean observed richness (the number of species detected). Bar represents 95% Bayesian credible intervals (Estimated) or confidence intervals (Observed). The 95% CIs of observed richness are very wide because of small sample size (n = 2).
(PDF)

S4 Fig Species responses to two environmental covariates. (a) landscape heterogeneity (from proportion of production agricultural lands to increasing SHDI) and (b) the amount of managed fields (CP38 fields) within a 1.5 km radius area surrounding a sample point. α_1_ and α_2_ are estimated from the multi-species dynamic occupancy model. Bars represent 95% Bayesian credible intervals. See S1 Table for species common name.
(PDF)

S1 Table Species and their traits used in analyses. (PDF)

S1 Appendix Codes used for hierarchical multi-species dynamic occupancy. To calculate Autocovariate (Acov), first we identified the distance of spatial autocorrelation (neighborhood size) for each of 15 species. Then, we calculated Acov in R 3.1 (“spdep” package), based on the product of occupancy status of neighbors of a site and inverse-Euclidian distance between the neighbors and a site. We calculated Acov based on the observed occupancy state (detection/non-detection data), whereas Royle and Dorazio (2008) used the latent occupancy state (from occupancy model).
(PDF)
