# Supplementary material for: Avian Species and Functional Diversity in Agricultural Landscapes: Does Landscape Heterogeneity Matter?
Source: PLoS One. 2017 Jan 26;12(1):e0170540. doi: 10.1371/journal.pone.0170540 (PMC5268393; doi:10.1371/journal.pone.0170540)

S1 Figure. The mean squared deviation (mSD) with varying numbers of dimensions (i.e., PCoA axes) of functional spaces and the scree plot showing the eigenvalue of each PCoA axis.

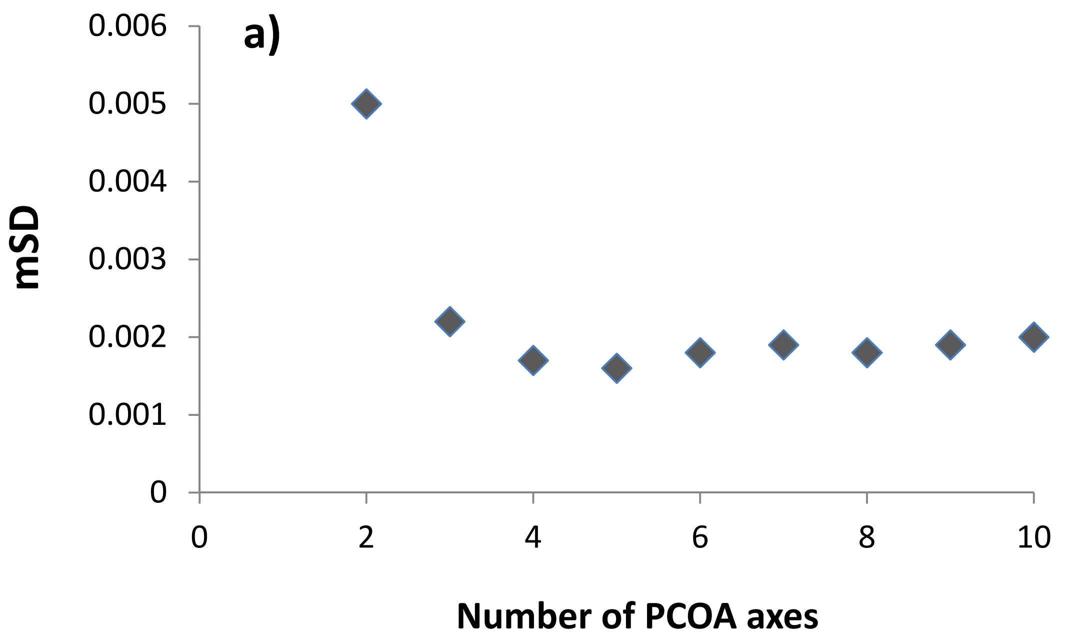


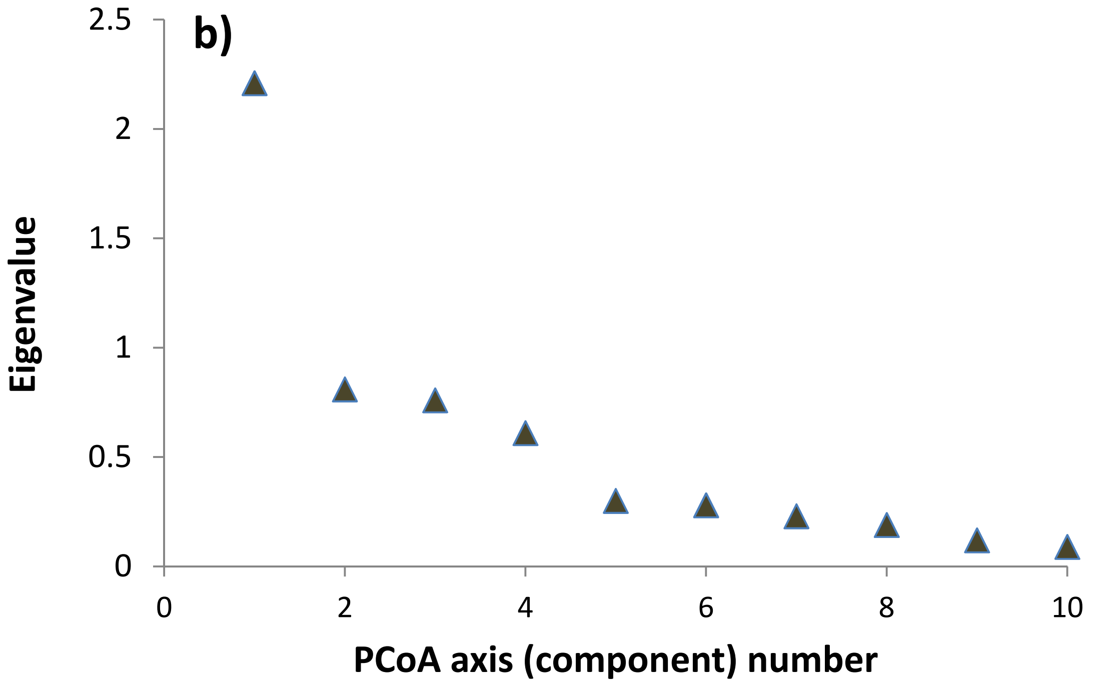

Supplement: S1 Fig — (PDF) [file pone.0170540.s001.pdf]
