# Supplementary figures and images for: Avian Species and Functional Diversity in Agricultural Landscapes: Does Landscape Heterogeneity Matter?
Source: PLoS One. 2017 Jan 26;12(1):e0170540. doi: 10.1371/journal.pone.0170540 (PMC5268393; doi:10.1371/journal.pone.0170540)

S2 Figure. Relationship between species richness and (a) FEve and (b) FDvi.

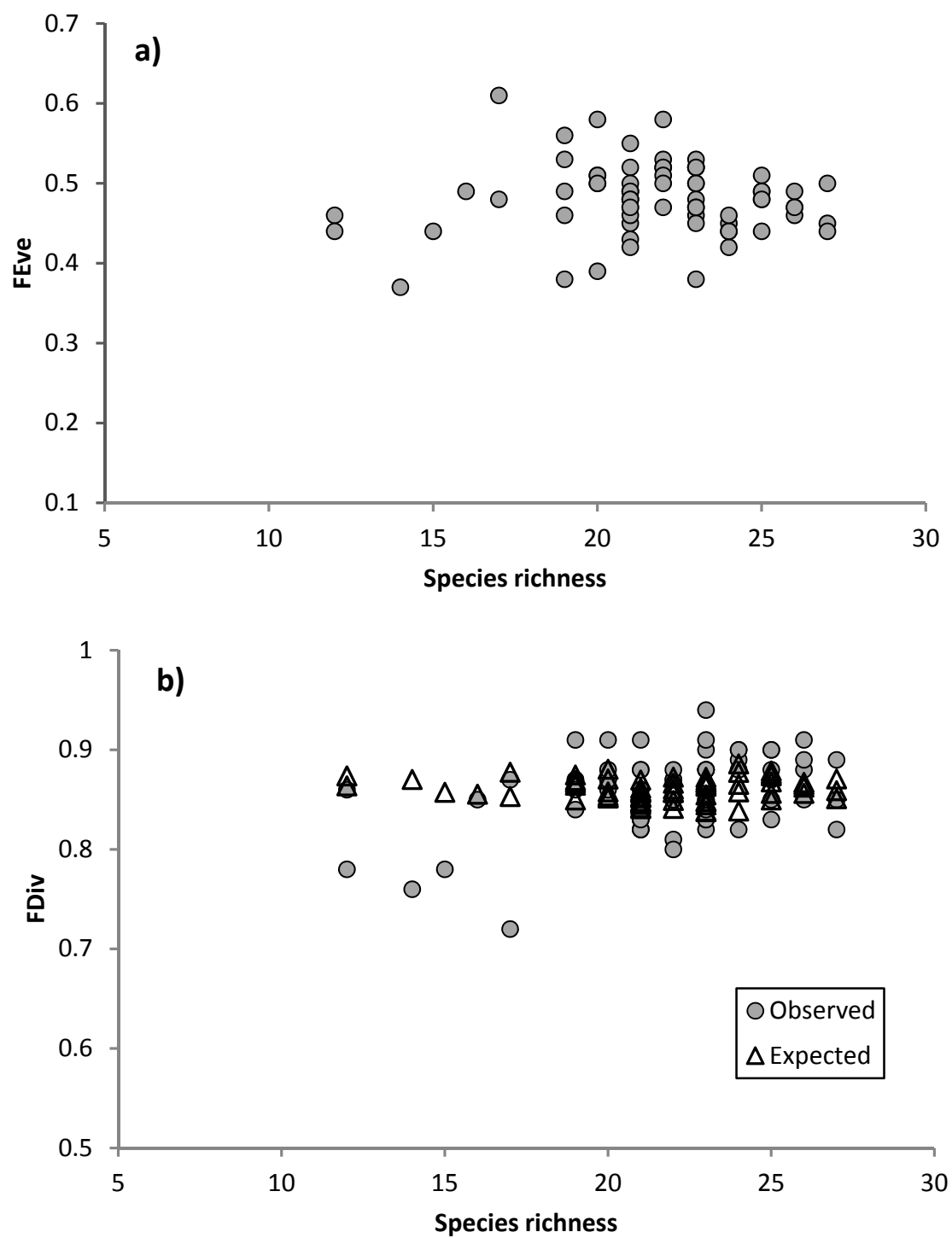

Supplement: S2 Fig — Expected FDiv is the mean FDiv value obtained from 999 random communities. (PDF) [file pone.0170540.s002.pdf]

S4 Figure. Species responses to two environmental covariates.

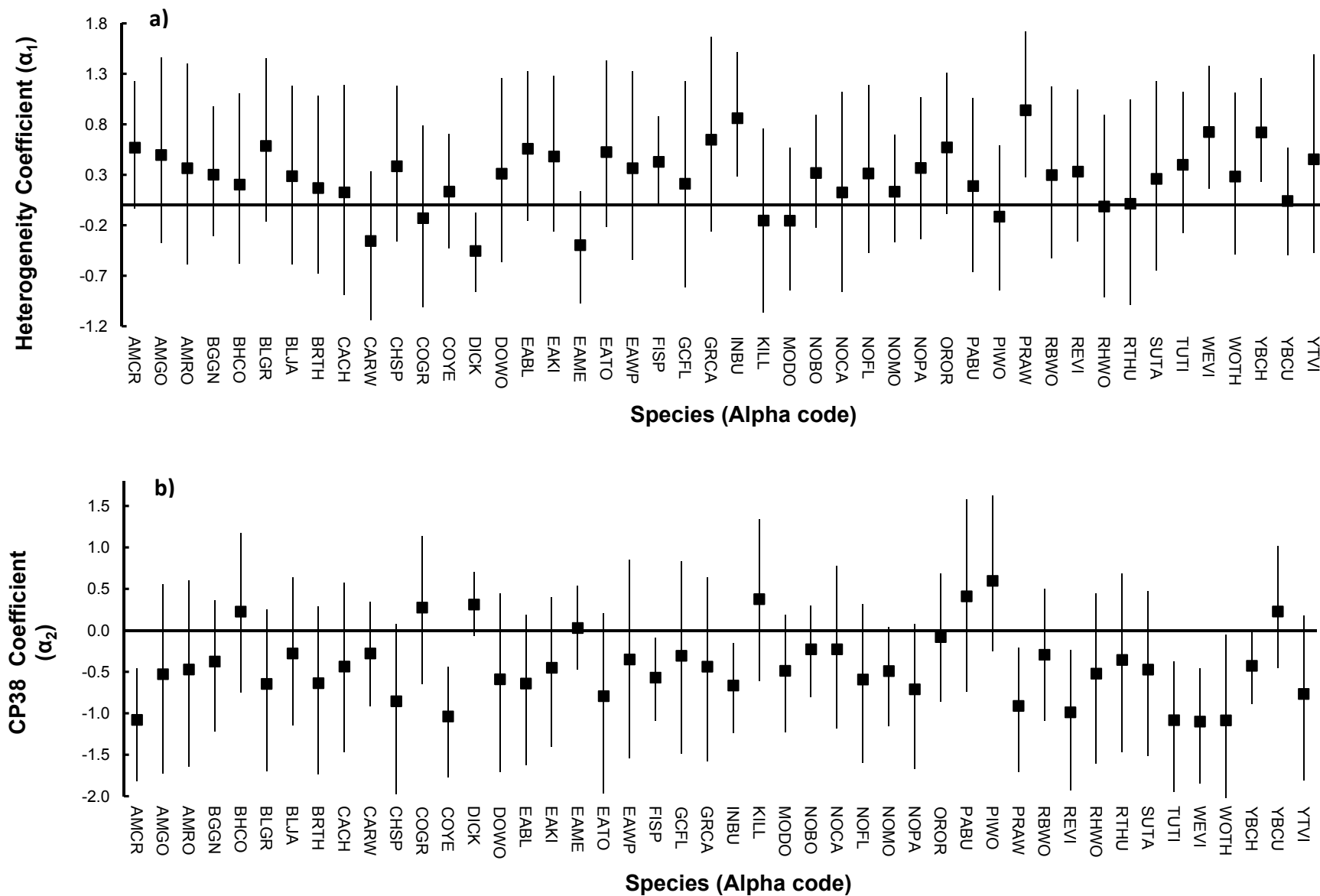

Supplement: S4 Fig — (a) landscape heterogeneity (from proportion of production agricultural lands to increasing SHDI) and (b) the amount of managed fields (CP38 fields) within a 1.5 km radius area surrounding a sample point. α1 and α2 are estimated from the multi-species dynamic occupancy model. Bars represent 95% Bayesian credible intervals. See S1 Table for species common name. (PDF) [file pone.0170540.s004.pdf]
