# Supplementary material for: Avian Species and Functional Diversity in Agricultural Landscapes: Does Landscape Heterogeneity Matter?
Source: PLoS One. 2017 Jan 26;12(1):e0170540. doi: 10.1371/journal.pone.0170540 (PMC5268393; doi:10.1371/journal.pone.0170540)

S3 Figure. Comparison of mean estimated richness (resulting from a hierarchical multi-species dynamic occupancy model) and observed richness (the number of species detected).

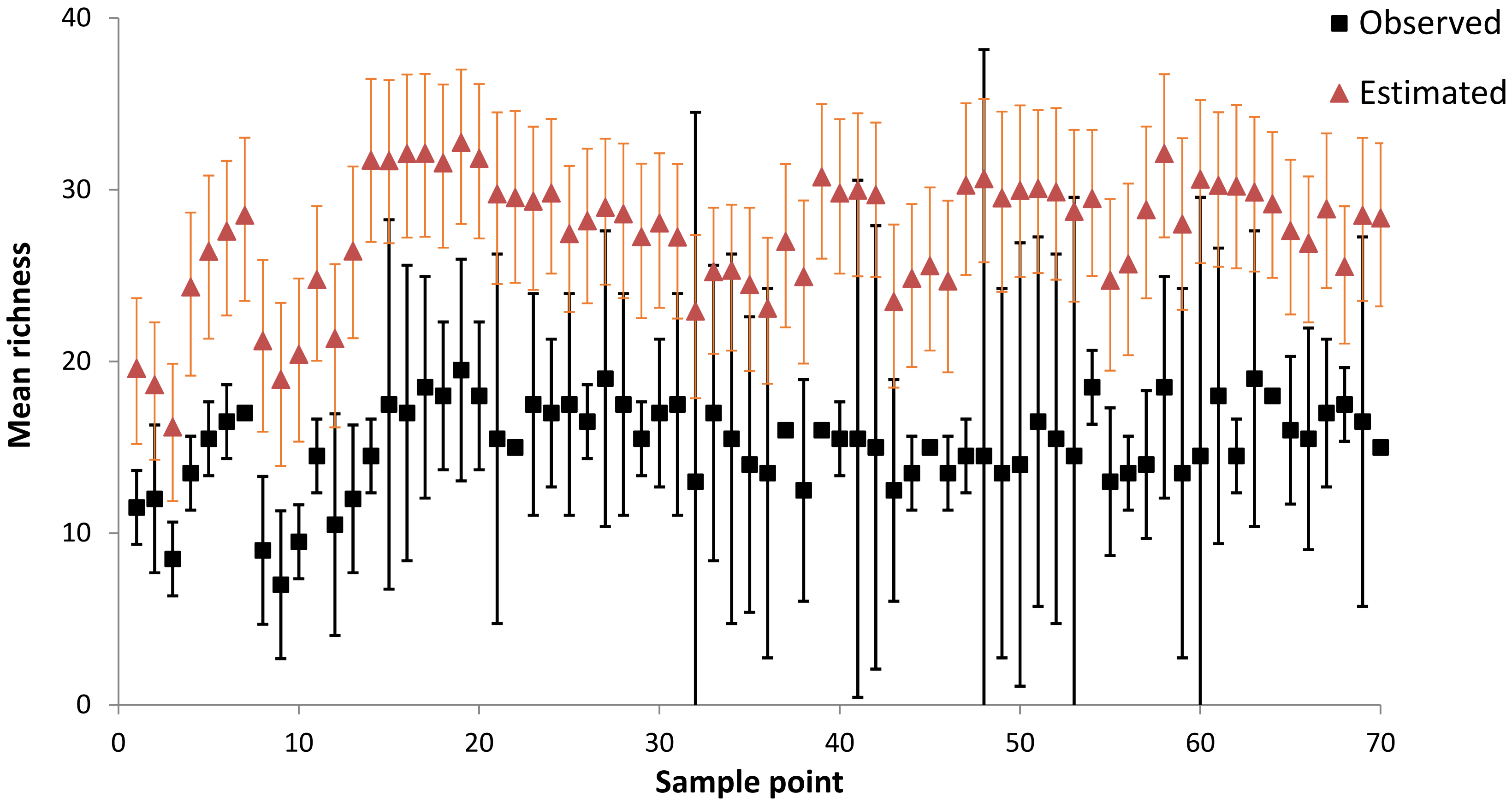

Supplement: S3 Fig — Bar represents 95% Bayesian credible intervals (Estimated) or confidence intervals (Observed). The 95% CIs of observed richness are very wide because of small sample size (n = 2). (PDF) [file pone.0170540.s003.pdf]
