# Supplementary material for: Avian Species and Functional Diversity in Agricultural Landscapes: Does Landscape Heterogeneity Matter?
Source: PLoS One. 2017 Jan 26;12(1):e0170540. doi: 10.1371/journal.pone.0170540 (PMC5268393; doi:10.1371/journal.pone.0170540)
Supplement: S1 Table — (PDF) [file pone.0170540.s005.pdf]

S1 Table. Species and their traits used in analyses.

Common name *Scientific name*	Alpha Code	Body Mass[1] (g)	Diet Type[2]				Foraging behavior and location[3]						Migration (Migrant)[4]	Habitat preference[5]
			DIn	DSg	DOm	DOt	FFg	FBg	FHw	FGf	FSa	FOt		
American Crow *Corvus brachyrhynchos*	AMCR	467	1	1	1	0	0	0	0	1	0	0	0	Fed
American Goldfinch *Spinus tristis*	AMGO	12.5	0	1	0	0	1	0	0	0	0	0	0	Shr
American Robin *Turdus migratorius*	AMRO	77.3	1	0	0	0	0	0	0	1	0	0	0	Fed
Blue-gray gnatcatcher *Polioptila caerulea*	BGGN	6	1	0	0	0	1	0	0	0	0	0	0	Fed
Brown-headed Cowbird *Molothrus ater*	BHCO	48.9	1	1	0	0	0	0	0	1	0	0	0	Fed
Blue Grosbeak *Passerina caerulea*	BLGR	27.5	1	1	0	0	0	0	0	1	0	0	1	Ope
Blue Jay *Cyanocitta cristata*	BLJA	70.53	1	1	1	0	0	0	1	1	0	0	0	Fed
Brown Thrasher *Toxostoma rufum*	BRTH	68.8	1	1	0	0	0	0	0	1	0	0	0	Shr
Carolina Chickadee *Poecile carolinensis*	CACH	10.8	1	0	0	0	1	0	0	0	0	0	0	Fin
Carolina Wren *Thryothorus ludovicianus*	CARW	18.6	1	0	0	0	0	0	0	1	0	0	0	Fed
Chipping Sparrow *Spizella passerina*	CHSP	12.3	1	1	0	0	0	0	0	1	0	0	1	Ope
Common Grackle *Quiscalus quiscula*	COGR	92.2	1	1	1	0	0	0	0	1	0	0	0	Fed
Common Yellowthroat *Geothlypis trichas*	COYE	9.9	1	0	0	0	1	0	0	0	0	0	1	Shr
Dickcissel *Spiza americana*	DICK	25.2	1	1	0	0	0	0	0	1	0	0	1	Ear[6]

Downy Woodpecker *Picoides pubescens*	DOWO	21.3	1	0	0	0	0	1	0	0	0	0	0	Fin
Eastern Bluebird *Sialia sialis*	EABL	30.5	1	0	0	0	0	0	0	0	1	0	0	Fed
Eastern Kingbird *Tyrannus tyrannus*	EAKI	41.6	1	0	0	0	0	0	1	0	0	0	1	Ope/Fed
Eastern Meadowlark *Sturnella magna*	EAME	82.3	1	0	0	0	0	0	0	1	0	0	0	Ear[6]
Eastern Towhee *Pipilo erythrophthalmus*	EATO	39.3	1	1	1	0	0	0	0	1	0	0	0	Shr
Eastern Wood-Pewee *Contopus virens*	EAWP	14.1	1	0	0	0	0	0	0	0	1	0	1	Ope
Field Sparrow *Spizella pusilla*	FISP	13	1	1	0	0	0	0	0	1	0	0	0	Ear[6]
Great Crested Flycatcher *Myiarchus crinitus*	GCFL	33.5	1	0	0	0	0	0	1	0	1	0	1	Ope
Gray Catbird *Dumetella carolinensis*	GRCA	39.6	1	0	0	0	0	0	0	1	0	0	1	Fed
Indigo Bunting *Passerina cyanea*	INBU	14.38	1	0	0	0	1	0	0	0	0	0	1	Ope
Killdeer *Charadrius vociferus*	KILL	101	1	0	0	1	0	0	0	1	0	0	0	Oth
Mourning Dove *Zenaida macroura*	MODO	123	0	1	0	0	0	0	0	1	0	0	0	Fed
Northern Bobwhite *Colinus virginianus*	NOBO	170	1	1	0	0	0	0	0	1	0	0	0	Ear[6]
Northern Cardinal *Cardinalis cardinalis*	NOCA	43	1	1	0	0	0	0	0	1	0	0	0	Fed/Shr
Northern Flicker *Colaptes auratus*	NOFL	129	1	0	0	0	0	0	0	1	0	1	0	Fed
Northern Mockingbird *Mimus polyglottos*	NOMO	47.2	1	0	0	0	0	0	1	1	1	0	0	Oth

Northern Parula *Parula americana*	NOPA	7.6	1	0	0	0	1	0	0	0	0	0	1	Fin
Orchard Oriole *Icterus spurius*	OROR	19.4	1	0	0	0	1	0	0	0	0	0	1	Ope
Painted Bunting *Passerina ciris*	PABU	15.2	1	1	0	0	0	0	0	1	0	0	1	Ope/Shr
Pileated Woodpecker *Dryocopus pileatus*	PIWO	234	1	0	0	0	0	1	0	0	0	0	0	Fin
Prairie Warbler *Dendroica discolor*	PRAW	7.7	1	0	0	0	1	0	0	0	0	0	1	Ope/Ear[6]
Red-bellied Woodpecker *Melanerpes carolinus*	RBWO	57.6	1	0	0	0	0	1	0	0	0	0	0	Fin
Red-eyed Vireo *Vireo olivaceus*	REVI	19.9	1	0	0	0	1	0	0	0	0	0	1	Fin
Red-headed Woodpecker *Melanerpes erythrocephalus*	RHWO	71.6	1	1	1	0	0	0	1	0	1	0	1	Ope
Ruby-throated Hummingbird *Archilochus colubris*	RTHU	3.81	1	0	0	1	0	0	0	0	0	1	1	Fed
Summer Tanager *Piranga rubra*	SUTA	28.87	1	0	0	0	1	0	1	0	0	0	1	Fin
Tufted Titmouse *Baeolophus bicolor*	TUTI	20.5	1	1	0	0	1	0	0	0	0	0	0	Fin
White-eyed Vireo *Vireo griseus*	WEVI	11.7	1	0	0	0	1	0	0	0	0	0	1	Fed/Shr
Wood Thrush *Hylocichla mustelina*	WOTH	50.1	1	0	0	0	0	0	0	1	0	0	1	Fin
Yellow-breasted Chat *Icteria virens*	YBCH	24.8	1	0	0	0	1	0	0	0	0	0	1	Fed/Shr
Yellow-billed Cuckoo *Coccyzus americanus*	YBCU	62.6	1	0	0	0	1	0	0	0	0	0	1	Ope
Yellow-throated Vireo *Vireo flavifrons*	YTVI	18	1	0	0	0	1	0	0	0	0	0	1	Fed

[1] Body mass is highly correlated with many other traits such as metabolic rate, foraging behavior, life time and home-range size (Flynn et al. 2009; Luck et al. 2012).
[2] Diet trait affects all aspects of foraging trait including behavior, method, location, food handling and processing (Flynn et al. 2009; Luck et al. 2012). Abbreviations of diet type: DIn, insects; DSg, seed/grass; DOm, omnivorous items; DOt, other diets including invertebrates other than insects and nectar
[3] Foraging (behavior and location) trait influences resource use by bird (Flynn et al. 2009; Luck et al. 2012). Abbreviations of foraging strategies: FFg, foliage gleaning; FBg, bark gleaning; FHw, hawking; FGf, ground foraging; FSa, sallying; FOt, other foraging methods such as probing and hovering. Sallying is similar to hawking (catching and feeding insects in the air), but it does not feed insects in the air. Instead, a bird returns to a perch site and feed insects after catching them in the air.
[4] Migratory status can influence “large-scale cycling of nutrients and the delivery of services of across broad regions" (Luck et al. 2012)” and it is largely determined by the activity period, main foraging period of a species (Calba et al. 2014).
[5] Habitat preference is based on the main habitat type used by a species for breeding. For instance, forest interior species (Fin) nest within the interior of the forest and avoid edge habitats. Early successional/grassland species (Ear) is species using early successional/grassland habitats for their breeding. Early successional/grassland habitats include grass- weedy areas (with forbs), grasslands, old farmlands, patchy shrublands, and very young forest. Open-forest species favor open habitats composed of a mixture of trees, shrubs, and grasses. Habitat requirements of some open-forest species overlap with those of early successional/grassland species. Abbreviation: Fin, forest interior; Fed, forest edge; Shr, shrub; Open, open-forest; Ear, early successional/grassland; Oth, others (mostly artificial structures such as houses).
[6]Species considered in CP38. Although the focal species CP38 was Northern Bobwhite, CP38 was also managed for other early successiona/grassland species.
