# Supplementary material for: Avian Species and Functional Diversity in Agricultural Landscapes: Does Landscape Heterogeneity Matter?
Source: PLoS One. 2017 Jan 26;12(1):e0170540. doi: 10.1371/journal.pone.0170540 (PMC5268393; doi:10.1371/journal.pone.0170540)
Supplement: S1 Appendix — To calculate Autocovariate (Acov), first we identified the distance of spatial autocorrelation (neighborhood size) for each of 15 species. Then, we calculated Acov in R 3.1 (“spdep” package), based on the product of occupancy status of neighbors of a site and inverse-Euclidian distance between the neighbors and a site. We calculated Acov based on the observed occupancy state (detection/non-detection data), whereas Royle and Dorazio (2008) used the latent occupancy state (from occupancy model). (PDF) [file pone.0170540.s006.pdf]

S1 Appendix. Codes used for hierarchical multi-species dynamic occupancy modeling.

```
model {

# Prior distributions for the community-level occupancy covariates and the detection covariates
alpha.mean ~ dunif(0,1)
alpha <- log(alpha.mean) - log(1-alpha.mean)

beta.mean ~ dunif(0,1)
beta <- log(beta.mean) - log(1-beta.mean)

mu.alpha1 ~ dunif(-5, 5)
mu.alpha2 ~ dunif(-5, 5)

sigma.alpha1 ~ dunif(0,5)
sigma.alpha2 ~ dunif(0,5)
tau.alpha1 <- pow(sigma.alpha1,-2)
tau.alpha2 <- pow(sigma.alpha2,-2)

sigma.u ~ dunif(0,10)
sigma.v ~ dunif(0,10)
tau.u <- pow(sigma.u,-2)
tau.v <- pow(sigma.v,-2)
rho ~ dunif(-1,1)
var.eta <- tau.v/(1.-pow(rho,2))


mu.delta ~ dunif(-5,5)
sigma.delta ~ dunif(0,5)
tau.delta <- pow(sigma.delta,-2)

kappa0 ~ dunif(0,1)
mu. kappa <- log(kappa 0/(1- kappa0))
sigma. kappa ~ dunif(0,10)
tau. kappa <- pow(sigma. kappa,-2)

upsilon0 ~ dunif(0,1)
mu. upsilon <- log(upsilon 0/(1- upsilon 0))
sigma. upsilon ~ dunif(0,10)
tau. upsilon <- pow(sigma. upsilon,-2)


#Prior distributions for the hyper-parameters for occupancy covariates and detection for each species
#i and n indexes species and number of species, respectively
```

```
 #rho indicates a correlation between occurrence and detection
 #delta is a coefficient of Autocovariate (Acov)
 #survival coefficient (kappa) and colonization coefficient (upsilon)


 for (i in 1:n) {

 u[i] ~ dnorm(alpha, tau.u)
 mu.eta[i] <- beta +(rho*sigma.v/sigma.u)*(u[i]-alpha)
 v[i] ~ dnorm(mu.eta[i], var.eta)

 alpha1[i] ~ dnorm(mu.alpha1, tau.alpha1)
 alpha2[i] ~ dnorm(mu.alpha2, tau.alpha2)

 delta[i] ~ dnorm(mu.delta, tau.delta)


kappa[i] ~ dnorm(mu.kappa, tau.kappa)
upsilon [i] ~ dnorm(mu. upsilon, tau. upsilon)

}


#Likelihood
 #j and J indexes site and number of sites, respectively
 #k indexes survey (replicate) within a year

 #Occupancy and detection model at year 1(t=1)

  for (i in 1:n) {

  for (j in 1:J) {

#Occupancy model (latent state)
 mean0[j,i,1] <- u[i] + alpha1[i]*Heterogeneity[j] + alpha2[i]*CP38[j] + delta[i]*Acov[j,i]
 mean1[j,i,1] <- 1/(1+exp(-mean0[j,i,1]))
 psi[j,i,1] <- max(0.0001,min(0.9999, mean1[j,i,1]))
 z[j,i,1] ~ dbin(psi[j,i,1], 1)


 for (k in 1:2) {

 #Detection model (observation model)
 mean2[j,i,1,k] <- v[i]
 theta[j,i,1,k] <- 1/(1+exp(-mean2[j,i,1,k]))
 mu.theta[j,i,1,k] <- theta[j,i,1,k]*z[j,i,1]
```

```
y[j,i,1,k] ~ dbin(mu.theta[j,i,1,k], 1)
ynew[j,i,1,k] ~ dbin(mu.theta[j,i,1,k], 1)
}
}
}


#Occupancy and detection model at year 2(t=2)
for (i in 1:46) {

for (j in 1:70) {

#Occupancy model (latent state)
mean0[j,i,2] <- u[i]  + z[j,i,1]*kappa[i] + (1-z[j,i,1])*upsilon[i] + alpha1[i]*heterogeneity[j] +
alpha2[i]*CP38 [j] + delta[i]*Acov[j,i]
mean1[j,i,2] <- 1/(1+exp(-mean0[j,i,2]))
psi[j,i,2] <- max(0.0001,min(0.9999, mean1[j,i,2]))
z[j,i,2] ~ dbin(psi[j,i,2], 1)

for (k in 1:2) {

#Detection model (observation model)
mean2[j,i,2,k] <- v[i]
theta[j,i,2,k] <- 1/(1+exp(-mean2[j,i,2,k]))
mu.theta[j,i,2,k] <- theta[j,i,2,k]*z[j,i,2]
y[j,i,2,k] ~ dbin(mu.theta[j,i,2,k], 1)
ynew[j,i,2,k] ~ dbin(mu.theta[j,i,2,k], 1)

}
}
}
# to estimate richness at each site and year
for (j in 1:J) {
for(t in 1:2) {
N[j,t] <- sum(z[j,,t])
}
}
# create simulate dataset to calculate the Bayesian p-value
for(i in 1:n){
for(j in 1:J){
for(t in 1:2){
for (k in 1:2){
d[j,i,t,k] <- abs(y[j,i,t,k]-mu.theta[j,i,t,k])
d2[j,i,t,k] <- pow(d[j,i,t,k],2)
dnew[j,i,t,k] <- abs(ynew[j,i,t,k]-mu.theta[j,i,t,k])
dnew2[j,i,t,k] <- pow(dnew[j,i,t,k],2)
```

```
}
dsum[j,i,t] <- sum(d2[j,i,t,1:2])
dnewsum[j,i,t] <-sum(dnew2[j,i,t,1:2])
}
}
}
p.fit <- sum(dsum[,,])
p.fitnew <- sum(dnewsum[,,])
b_pvalue <- step(p.fitnew-p.fit)
}
```
